# Myelodysplastic syndromes complicated by atypical Sweet syndrome: a brief research report

**DOI:** 10.3389/fmed.2026.1839456

**Published:** 2026-05-28

**Authors:** Bianhong Wang, Kun Qian, Hao Liu, Yuehua Huang, Yanying Wang, Lihong Li, Fan Yu

**Affiliations:** 1 Department of Hematology, School of Clinical Medicine, Tsinghua Medicine, Beijing Tsinghua Changgung Hospital, Tsinghua University, Beijing, China; 2 Department of Hematology, The Fifth Medical Center of Chinese PLA General Hospital, Beijing, China; 3 Department of Pathology, School of Clinical Medicine, Tsinghua Medicine, Beijing Tsinghua Changgung Hospital, Tsinghua University, Beijing, China

**Keywords:** hematologic malignancy, myelodysplastic syndromes, neutrophilic dermatosis, paraneoplastic syndrome, Sweet syndrome

## Abstract

**Objective:**

This study aimed to characterize the clinicopathological features and therapeutic outcomes of atypical Sweet syndrome (SS) in patients with myelodysplastic syndromes (MDS).

**Methods:**

A retrospective analysis was conducted on three patients diagnosed with MDS complicated by atypical SS at Beijing Tsinghua Changgung Hospital between 2019 and 2020.

**Results:**

The cohort consisted of a 58-year-old man, a 35-year-old woman, and a 46-year-old man. All patients initially presented with fatigue, and none had received active disease-modifying therapy for MDS prior to the onset of cutaneous manifestations. In all cases, skin lesions originated on the limbs, presenting as painful erythematous plaques accompanied by fever, with histopathological findings consistent with atypical SS. Resolution of SS was achieved through a dual approach combining systemic glucocorticoids and therapy targeting the underlying MDS.

**Conclusion:**

Atypical SS is a significant paraneoplastic dermatosis frequently associated with MDS, exhibiting distinctive clinicopathological features. Its heterogeneous presentations necessitate careful evaluation, and effective management requires a dual strategy that addresses both the underlying hematologic disease and the cutaneous inflammation.

## Introduction

Sweet syndrome (SS), also known as acute febrile neutrophilic dermatosis, was first described by Robert Sweet in 1964 (1). It is characterized by acute fever, tender erythematous plaques or nodules, and dense dermal neutrophilic infiltration without vasculitis on histopathology (1, 2). SS is classified into three subtypes: classical (idiopathic) SS, malignancy-associated SS, and drug-induced SS (2, 3). Diagnosis is based on the revised criteria proposed by von den Driesch in 1994, which include two major and at least two minor criteria (3).

SS is strongly associated with malignancy, acting as a paraneoplastic syndrome that may precede, coincide with, or indicate recurrence of an underlying neoplasm (4). Approximately 85% of malignancy-associated SS cases are associated with hematologic disorders, most commonly acute myeloid leukemia (AML) and myelodysplastic syndromes (MDS) (5). However, focused clinical studies of MDS-associated atypical SS remain limited. In this study, we present three cases of MDS complicated by atypical SS, along with a literature review, to improve recognition, diagnosis, and integrated management (6, 7).

## Patients and methods

This was a retrospective, single-center case series conducted at the Department of Hematology of Beijing Tsinghua Changgung Hospital. Three patients diagnosed with MDS complicated by SS between 2019 and 2020 were enrolled.

### Inclusion criteria

The inclusion criteria were as follows:

1. Diagnosis of MDS confirmed by bone marrow morphology, immunophenotyping, cytogenetics, and molecular genetics (MICM) according to the 2016 World Health Organization (WHO) classification.

2. Diagnosis of SS confirmed by the revised criteria of Wolfgang von den Driesch (3).

3. Availability of complete clinical, laboratory, histopathological, treatment, and follow-up data.

Clinical data, including demographic information, symptoms, laboratory results, histopathology, treatment, and outcomes, were systematically collected and analyzed.

### Diagnostic criteria for SS (3)

#### Major criteria

The major criteria for SS include the following:

1. The sudden onset of painful erythematous plaques or nodules, occasionally with vesicles, pustules, or bullae.
2. Dense dermal neutrophilic infiltration without leukocytoclastic vasculitis on histopathology.

#### Minor criteria

The minor criteria for SS include the following:

1. Association with an underlying condition, such as hematologic or solid malignancy, inflammatory disorder, pregnancy, recent infection, or vaccination.
2. Fever >38 °C.
3. Prompt response to systemic glucocorticoids or potassium iodide.
4. At least three of the following: erythrocyte sedimentation rate (ESR) > 20 mm/h, elevated C-reactive protein (CRP), white blood cell (WBC) count >8.0 × 10^9^/L, and neutrophils >70%.

Diagnosis requires both major criteria and at least two minor criteria.

## Results

Three patients were included in the study (Table 1). All patients presented with fatigue as the initial MDS symptom. None received active MDS-modifying therapy at the time of SS onset.

**Table 1:** Clinical characteristics of three patients with MDS complicated by SS.

Items	Sex/age	Initial symptoms of MDS	Complete blood count at SS onset	MDS subtypes (blasts %)	Cytogenetics	Gene mutations	MDS treatments before SS onset	SS cutaneous features	SS clinical manifestations	SS treatments	Clinical outcomes
Case 1	Male, 58 years	Fatigue	WBC 4.32 × 10^9/L, Hgb 88 g/L, and PLT 395 × 10^9/L	MDS-EB-1 (7.5%)	Complex karyotype	None	1 × azacitidine + TCM	Limbs, then involving the head and neck	Plaques, ulcers, exudate, and intermittent fever	Dexamethasone + AZA + CAG	SS resolved, no recurrence
Case 2	Female, 35 years	Fatigue, fever	WBC 1.19 × 10^9/L, Hgb 58 g/L, and PLT 29 × 10^9/L	MDS-EB-2 (11%)	46, XX[20] (normal)	ASXL1 mutation	Supportive transfusions	Lower limbs	Cellulitis-like plaques, ulceration, and recurrent fever	Antibiotics→ allogeneic HSCT	SS resolved post-HSCT
Case 3	Male, 46 years	Fatigue	WBC 2.6 × 10^9/L, Hgb 72 g/L, and PLT 68 × 10^9/L	MDS-EB-2 (13%)	46, XY[20] (normal)	TET2 mutation	Azacitidine (progressed to AML)	Lower limbs	Cellulitis-like plaques, nodules, and recurrent fever	Methylprednisolone → allogeneic HSCT	SS improved; died of post-HSCT complications

MDS, myelodysplastic syndromes; SS, Sweet syndrome; CBC, complete blood count (×10^9/L for WBC/PLT, g/L for Hgb); EB, excess blasts; TCM, traditional Chinese medicine; AML, acute myeloid leukemia; HSCT, hematopoietic stem cell transplantation; AZA + CAG, azacitidine + cytarabine/aclacinomycin/granulocyte colony-stimulating factor (G-CSF) regimen.

To rule out infectious etiologies, all patients underwent cutaneous swab cultures for bacteria and fungi, as well as serological tests for common pathogens; all results were negative.

### Case 1

A 58-year-old man was diagnosed with myelodysplastic syndrome with excess blasts-1 (MDS-EB-1, 7.5% blasts), a complex karyotype, and no gene mutations. Before SS onset, he received one cycle of azacitidine and traditional Chinese medicine (TCM) as supportive care to improve fatigue and cytopenias, with no anti-neoplastic effect.

He presented with painful erythematous plaques on the extremities, extending to the head and neck, with central ulceration, exudation, and intermittent fever.

At SS onset, the white blood cell (WBC) count was 4.32 × 10^9^/L, the hemoglobin (Hgb) level was 88 g/L, and the platelet (PLT) count was 395 × 10^9^/L.

**Figure 1:**
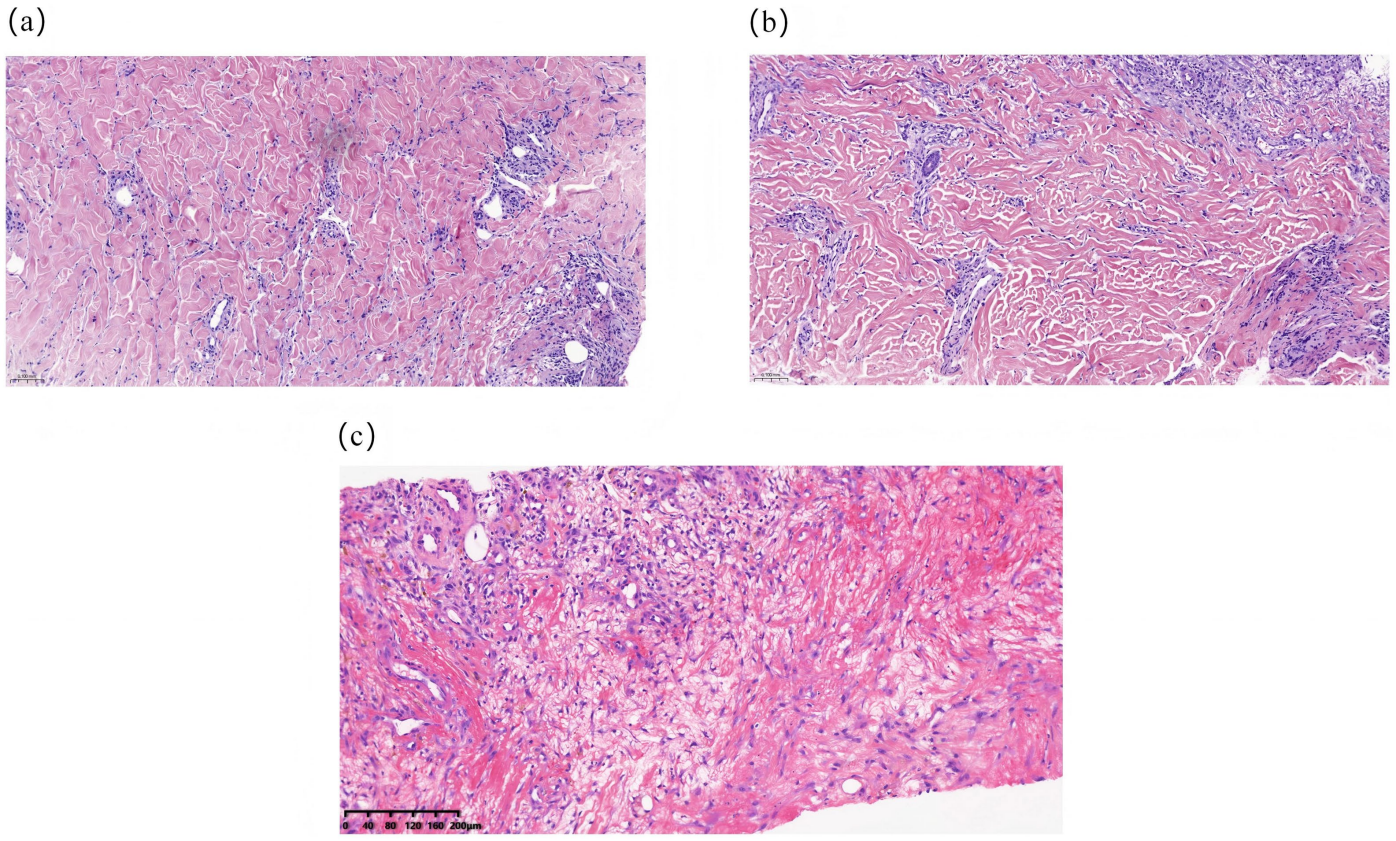
Histopathological findings of skin tissues in three patients (HE staining). **(a–c)** Correspond to Case 1, Case 2, and Case 3, respectively. The epidermis is well preserved, and all three patients exhibited epidermal hyperkeratosis, prominent dermal edema, and dense perivascular neutrophilic infiltration in the superficial dermis, accompanied by scattered lymphocytes and histiocytes. No leukocytoclastic vasculitis was observed (HE staining, ×100).

A skin biopsy showed epidermal hyperkeratosis, marked dermal edema, and dense perivascular neutrophilic infiltration without leukocytoclastic vasculitis (Figure 1a).

**Figure 2:**
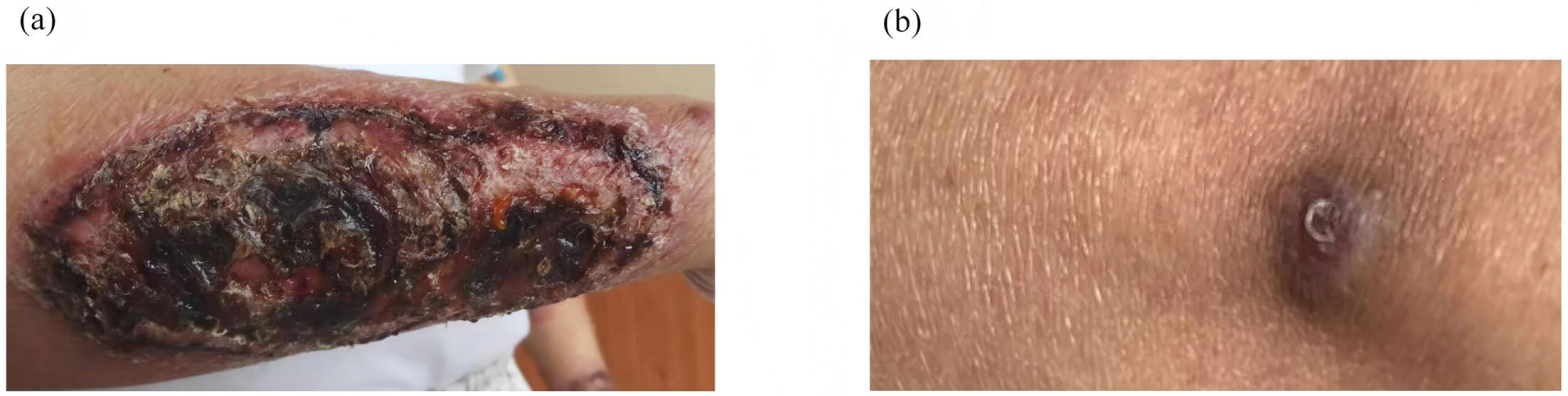
Skin manifestations of Case 1. **(a)** Before treatment. **(b)** After treatment.

He was treated with dexamethasone combined with an azacitidine + cytarabine/aclacinomycin/granulocyte colony-stimulating factor (G-CSF) regimen (AZA + CAG) chemotherapy. Fever and skin lesions resolved completely without recurrence (Figure 2b).

### Case 2

A 35-year-old woman was diagnosed with MDS-EB-2 (11% blasts), a normal karyotype, and an additional sex combs-like 1 (ASXL1) mutation. Before SS onset, only supportive transfusions were given.

She developed cellulitis-like painful plaques on the lower limbs with ulceration and recurrent high fever.

At SS onset, the white blood cell (WBC) count was 1.19 × 10^9^/L, the hemoglobin (Hgb) level was 58 g/L, and the platelet (PLT) count was 29 × 10^9^/L.

**Figure 3:**
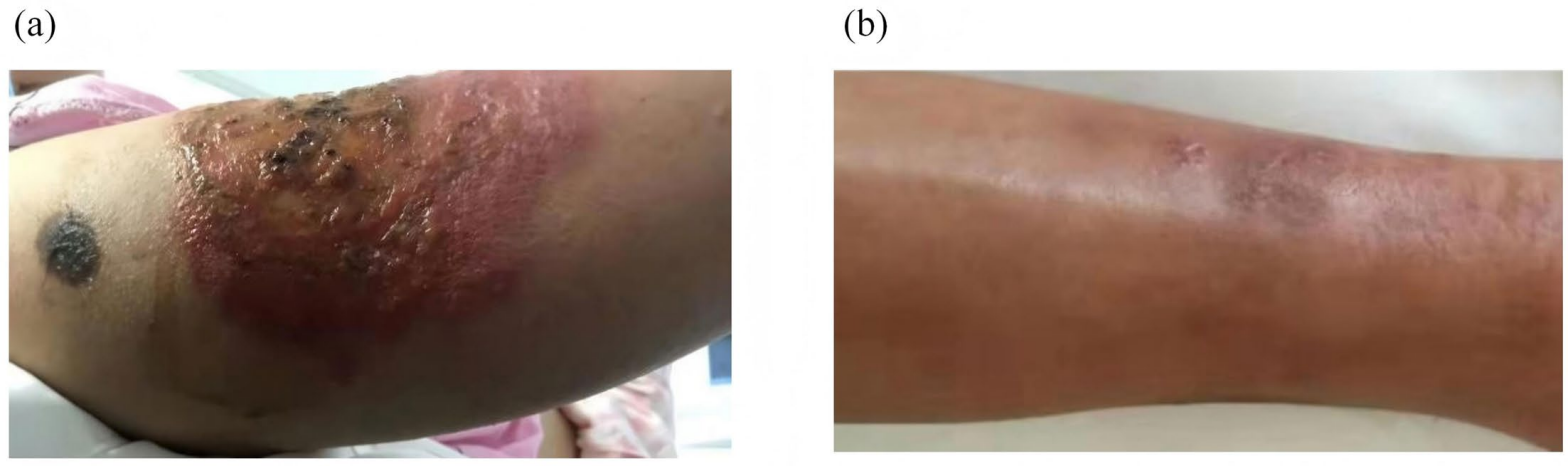
Skin manifestations of Case 2. **(a)** Before treatment. **(b)** After treatment.

A skin biopsy showed dermal neutrophilic infiltration consistent with atypical SS (Figure 1b). Antibiotics resulted in only temporary improvement. She underwent allogeneic hematopoietic stem cell transplantation (HSCT), and SS resolved completely after transplantation (Figure 3b).

### Case 3

A 46-year-old man was diagnosed with MDS-EB-2 (13% blasts), a normal karyotype, and a ten-eleven translocation-2 (TET2) mutation. He had received azacitidine and progressed to acute myeloid leukemia (AML) before SS onset.

He presented with painful subcutaneous nodules and erythematous plaques on the lower limbs, accompanied by recurrent fever.

At SS onset, the white blood cell (WBC) count was 2.6 × 10^9^/L, the hemoglobin (Hgb) level was 72 g/L, and the platelet (PLT) count was 68 × 10^9^/L.

A skin biopsy confirmed atypical SS. He was treated with methylprednisolone with rapid improvement. He later received allogeneic HSCT but died of transplant-related complications.

### Histopathological and laboratory findings

Skin biopsies showed epidermal hyperkeratosis, marked dermal edema, and dense perivascular neutrophilic infiltration in the superficial dermis, with scattered lymphocytes and histiocytes. No leukocytoclastic vasculitis was observed (HE staining, × 100) (Figure 1).

At SS onset:

- Case 1: WBC: 4.32 × 10^9/L, Hgb: 88 g/L, and PLT: 395 × 10^9/L
- Case 2: WBC: 1.19 × 10^9/L, Hgb: 58 g/L, and PLT: 29 × 10^9/L
- Case 3: WBC: 2.6 × 10^9/L, Hgb: 72 g/L, and PLT: 68 × 10^9/L

Bone marrow and molecular features confirmed MDS-EB-1 or MDS-EB-2. Case 1 had a complex karyotype and Cases 2 and 3 had normal karyotypes with an ASXL1 and a TET2 mutation, respectively.

### Treatment and outcomes

Case 1: Following MDS diagnosis, the patient received decitabine (20 mg/day, days 1–5), resulting in grade IV myelosuppression. Subsequent treatment was switched to traditional Chinese medicine. Ten months later, Sweet syndrome (SS) developed. Bone marrow examination revealed 44% blasts. The patient was treated with dexamethasone (10 mg/day, tapered gradually) and a one cycle of AZA + CAG chemotherapy [azacitidine 135 mg/day, days 1–7; granulocyte colony-stimulating factor (G-CSF) from day 6; aclarubicin 18 mg/day, days 7–11; and cytarabine 18 mg Q12H, days 7–13]. The fever resolved, skin lesions healed without recurrence, and glucocorticoids were successfully discontinued (Figure 2).

Case 2: After MDS diagnosis, the patient received supportive care with red blood cell and platelet transfusions and was scheduled for allogeneic hematopoietic stem cell transplantation (allo-HSCT). Skin lesions were initially managed as infections with vancomycin, leading to transient improvement; however, symptoms recurred upon discontinuation. SS was subsequently suspected. The patient underwent successful allo-HSCT and remained free of skin lesion recurrence during follow-up (Figure 3).

Case 3: Post-MDS diagnosis, the patient received DCAG chemotherapy (decitabine 38 mg/day, days 1–5; aclarubicin 20 mg/day, days 1–5; cytarabine 100 mg/day, days 1–5; and G-CSF from day 0 until neutrophil recovery) but progressed to AML. SS-like subcutaneous nodules and rash appeared on the right calf. Pathology confirmed SS. Methylprednisolone (initially 40 mg Q12H, tapered from March 5 to March 30) led to rapid defervescence and rash resolution. Allo-HSCT from a matched sibling donor was performed thereafter; however, the patient died of post-transplant pulmonary infection and cardiac failure.

## Discussion

Sweet syndrome represents a prototypical neutrophilic dermatosis whose pathogenesis, particularly in the context of malignancy, remains multifactorial and incompletely elucidated. According to recent literature, the prevalence of SS in patients with MDS ranges from 1.2 to 3.5%, representing a rare but clinically significant paraneoplastic complication.

Several interlinked mechanisms have been proposed. First, dysregulated cytokine signaling, notably elevated granulocyte colony-stimulating factor (G-CSF) levels commonly observed in myeloid neoplasms, may promote neutrophil proliferation, maturation, and cutaneous trafficking (8–10). This is supported by the clinical observations of SS development following exogenous G-CSF administration (8, 9). Second, SS in MDS is recognized as a reactive immune disorder resulting from a dysregulated inflammatory response rather than direct leukemic cell infiltration into the skin (11–14). Third, SS may represent a paraneoplastic hypersensitivity reaction, wherein tumor antigens or secreted cytokines trigger a localized inflammatory cascade and a cytokine storm (15). This concept aligns with the rapid response to immunosuppressive therapy but lacks consistent serological correlates, such as immune complex deposition. Fourth, malignant myeloid cells can alter the dermal microenvironment by upregulating endothelial adhesion molecules (e.g., selectins and integrins) and secreting pro-inflammatory mediators such as tumor necrosis factor-alpha (TNF-*α*) and interleukin-1 beta (IL-1β), thereby facilitating neutrophil extravasation and tissue infiltration (16, 17) These mechanisms are not mutually exclusive and may coexist in individual patients. Collectively, these mechanisms underscore the intricate interplay between the dysregulated myeloid clone and the host’s inflammatory response in driving SS pathogenesis (6, 18). The pathogenesis of SS involves dysregulated immune and inflammatory pathways rather than direct clonal malignant cell infiltration (14).

In our case series, all three patients presented with atypical cutaneous manifestations, including ulcerative, cellulitis-like, or nodular lesions that could easily be misdiagnosed as bacterial infections or other inflammatory skin conditions. Although the lesions were atypical, the diagnosis of SS was firmly confirmed by:

(1) Fulfillment of the von den Driesch criteria.
(2) Dense dermal neutrophilic infiltration without blast cells.
(3) Rapid response to glucocorticoids.
(4) No evidence of leukemia cutis.

This combination of clinical, histopathological, and therapeutic evidence reliably ruled out leukemia cutis and other mimickers, ensuring an accurate diagnosis even in the setting of atypical presentation.

Our case series reinforces the recognized association between SS and myeloid malignancies, particularly MDS (19). Notably, all three patients developed SS during periods of suboptimal disease control or progression, underscoring the parallel activity of SS and the underlying hematologic disorder. This temporal relationship highlights the importance of treating the primary malignancy as a fundamental component of SS management. In clinical practice, SS may occasionally serve as the initial presenting sign of an undiagnosed MDS or AML, particularly in patients with unexplained cytopenias and no typical rheumatologic features (20). Therefore, in patients presenting with new-onset SS, particularly those with constitutional symptoms or abnormal blood counts, a thorough hematologic evaluation, including bone marrow examination, should be considered to rule out an underlying myeloid neoplasm.

Clinically, SS exhibits considerable morphological variability beyond the classic plaques. In our series, all three patients presented with atypical ulcerative or cellulitis-like lesions, which are easily misdiagnosed as infection, pyoderma gangrenosum (PG), Behçet disease, or other inflammatory dermatoses. Early recognition and pathological confirmation are critical to avoid delayed treatment. As illustrated in our series, presentations may include pustular, bullous, ulcerative, or cellulitis-like morphologies (4), which can mimic infectious or other inflammatory dermatoses, leading to diagnostic delay. Moreover, extracutaneous involvement—affecting ocular, pulmonary, cardiac, gastrointestinal, or nervous systems—occurs in a subset of patients and may be associated with significant morbidity or mortality (4). Therefore, a high degree of clinical suspicion and multidisciplinary evaluation are essential for timely diagnosis and management.

Pyoderma gangrenosum (PG) and Adamantiades–Behçet disease (AB) are two key differential diagnoses to be distinguished from atypical Sweet syndrome, particularly in patients with MDS. PG typically presents with progressive, painful, undermined ulcers with violaceous borders (21), and histopathology lacks the dense dermal neutrophilic infiltration typical of SS. AB typically presents with recurrent oral aphthous ulcers, genital ulcers, uveitis, pustular skin lesions, and vascular involvement (22). In our three cases, we carefully confirmed that none of the patients had oral aphthous ulcers, genital ulcers, uveitis, or systemic vascular involvement. Histopathologically, leukocytoclastic vasculitis is a characteristic feature of AB; however, it was absent in all of our skin biopsy specimens. In addition, all skin lesions showed a rapid and complete response to systemic glucocorticoid therapy, which is a typical feature of Sweet syndrome rather than PG or Behçet disease. Therefore, PG and AB were reliably ruled out, and the diagnosis of atypical Sweet syndrome was firmly established (4).

Therapeutic strategies for malignancy-associated SS prioritize control of the underlying neoplasm alongside targeted anti-inflammatory treatment. Systemic glucocorticoids remain first-line therapy, typically initiated at 0.5–1 mg/kg/day prednisone equivalent and tapered gradually over 4–6 weeks upon clinical response (19). For patients with contraindications to steroids or refractory disease, alternative agents such as colchicine, potassium iodide, dapsone, cyclosporine, or biologic therapies, including TNF-*α* inhibitors, may be considered (19, 23). Importantly, clinicians should be aware that several medications commonly used in hematology—including G-CSF, cytarabine, hypomethylating agents, and FMS-like tyrosine kinase 3 (FLT3) inhibitors—have been implicated in drug-induced SS (4). This variant typically resolves upon drug discontinuation and responds well to steroids, highlighting the importance of a detailed medication history and etiological distinction.

In our cohort, therapeutic outcomes varied with the approach to the underlying MDS. The patient who achieved sustained hematologic remission through HSCT (Case 2) also experienced durable resolution of SS, whereas the patient who succumbed to transplant-related complications (Case 3) exhibited only transient dermatological improvement. These observations suggest that the long-term control of SS may be closely tied to the effective management of the associated malignancy.

## Limitations

This study is limited by its retrospective, single-center design and small sample size (*n* = 3), which may reduce its generalizability. Special stains, including periodic acid–Schiff (PAS) and Gram staining for bacteria or fungi, were not performed in skin specimens. We did not perform clonality analysis on skin lesions to confirm the molecular link between cutaneous infiltrates and bone marrow blasts. Treatment was not standardized due to the rarity of the condition. Further large-scale prospective studies are needed.

## Conclusion

Atypical Sweet syndrome is a clinically significant neutrophilic dermatosis that frequently accompanies myeloid malignancies, such as MDS. Its presentations are protean and may involve multiple organ systems, necessitating a comprehensive diagnostic approach. For patients with MDS presenting with unexplained fever and painful erythematous plaques, a prompt skin biopsy is essential to confirm atypical SS. Effective management requires a dual strategy addressing both the underlying hematologic disease and the cutaneous inflammatory process, with systemic glucocorticoids serving as the cornerstone of initial therapy. Enhanced clinical awareness and timely recognition of this paraneoplastic syndrome are crucial for optimizing patient outcomes through integrated hematologic and dermatologic care.

## Data Availability

The original contributions presented in the study are included in the article/supplementary material, further inquiries can be directed to the corresponding author.

## References

[1] Sweet R. An acute febrile neutrophilic dermatosis. Br J Dermatol. (1964) 76:349–56. doi: 10.1111/j.1365-2133.1964.tb14541.x, 14201182

[2] Heath MS Ortega-Loayza AG. Insights into the pathogenesis of Sweet's syndrome. Front Immunol. (2019) 10:414. doi: 10.3389/fimmu.2019.00414, 30930894 PMC6424218

[3] von den Driesch P. Sweet’s syndrome (acute febrile neutrophilic dermatosis). J Am Acad Dermatol. (1994) 31:535–56. doi: 10.1016/S0190-9622(94)70215-2 8089280

[4] Nelson CA Stephen S Ashchyan HJ James WD Micheletti RG Rosenbach M. Neutrophilic dermatoses: pathogenesis, Sweet syndrome, neutrophilic eccrine hidradenitis, and Behçet disease. J Am Acad Dermatol. (2018) 79:987–1006. doi: 10.1016/j.jaad.2017.11.064, 29653210

[5] Cohen PR Talpaz M Kurzrock R. Malignancy-associated Sweet's syndrome: review of the world literature. J Clin Oncol. (1988) 6:1887–97. doi: 10.1200/JCO.1988.6.12.1887, 3058878

[6] Ferea CR Mihai SN Balan G Badescu MC Tutunaru D Tatu AL. Sweet syndrome associated with myelodysplastic syndrome—a review of a multidisciplinary approach. Life. (2024) 13:809. doi: 10.3390/life13030809 PMC10053503 36983964

[7] Gil-Lianes MJ Luque-Luna M Alamon-Reig F Bosch-Amate X Serra-Garcia L Mascaró JM. Sweet syndrome: clinical presentation, malignancy association, autoinflammatory disorders and treatment response in a cohort of 93 patients with long-term follow-up. Acta Derm Venereol. (2025) 105:18284. doi: 10.2340/actadv.v103.18284 PMC10753595 38112209

[8] Nelson CA Noe MH McMahon CM McMahon MG Rosenbach M. Sweet syndrome in patients with and without malignancy: a retrospective analysis of 83 patients from a tertiary academic referral center. J Am Acad Dermatol. (2018) 78:303–309.e4. doi: 10.1016/j.jaad.2017.09.013, 29107342

[9] Magro CM De Moraes E Burns F. Sweet's syndrome in the setting of CD34-positive acute myelogenous leukemia treated with granulocyte colony stimulating factor: evidence for a clonal neutrophilic dermatosis. J Cutan Pathol. (2001) 28:90–6. doi: 10.1034/j.1600-0560.2001.280205.x, 11168757

[10] Sujobert P Cuccuini W Vignon-Pennamen MD Vignon-Pennamen D Martin-Garcia N Albertini AF . Evidence of differentiation in myeloid malignancies associated neutrophilic dermatosis: a fluorescent in situ hybridization study of 14 patients. J Invest Dermatol. (2013) 133:1111–4. doi: 10.1038/jid.2012.408, 23190893

[11] Van Loon K Gill RM McMahon P Litzner C Abraham T. 20q- clonality in a case of oral Sweet syndrome and myelodysplasia. Am J Clin Pathol. (2012) 137:310–5. doi: 10.1309/AJCP9I7NRWYLTJHV, 22261459

[12] Mo W Wang X Wang Y Zhang L Chen S. Clonal neutrophil infiltrates in concurrent Sweet’s syndrome and acute myeloid leukemia: a case report and literature review. Cancer Genet. (2018) 226-227:11–6. doi: 10.1016/j.cancergen.2018.04.120, 30005849

[13] Voelter-Mahlknecht S Bauer J Metzler G Hossfeld DK. Bullous variant of Sweet’s syndrome. Int J Dermatol. (2005) 44:946–7. doi: 10.1111/j.1365-4632.2004.02287.x, 16336530

[14] Calabrese L Romagnuolo M D'Onghia M Rubegni P Marzano AV Moltrasio C. Molecular characteristics of Sweet syndrome: a systematic review. Exp Dermatol. (2016) 33:e70022–508. doi: 10.1111/exd.70022, 39704328 PMC11660222

[15] Lyck R Enzmann G. The physiological roles of ICAM-1 and ICAM-2 in neutrophil migration into tissues. Curr Opin Hematol. (2015) 22:53–9. doi: 10.1097/MOH.0000000000000103, 25427141

[16] Stucki A Rivier AS Gikic M Monai N Schapira M Spertini O. Endothelial cell activation by myeloblasts: molecular mechanisms of leukostasis and leukemic cell dissemination. Blood. (2001) 97:2121–9. doi: 10.1182/blood.V97.7.2121, 11264180

[17] Marcoval J Martin-Callizo C Valenti-Medina F Bonfill‐Ortí M Martínez‐Molina L. Sweet syndrome: long-term follow-up of 138 patients. Clin Exp Dermatol. (2016) 41:741–6. doi: 10.1111/ced.12899, 27663147

[18] Weiss EH Ko CJ Leung TH . Neutrophilic Dermatoses: a Clinical Update. Curr Dermatol Rep. (2022) 11:89–102. doi: 10.1007/s13671-022-00355-8 35310367 PMC8924564

[19] Cohen PR. Neutrophilic dermatoses: a review of current treatment options. Am J Clin Dermatol. (2009) 10:301–12. doi: 10.2165/11310730-000000000-00000, 19658442

[20] Joseph S Hitawala AA Cohen B Falloon K Simonson M. Systematic review: Sweet syndrome associated with inflammatory bowel disease. J Crohns Colitis. (2021) 15:1864–76. doi: 10.1093/ecco-jcc/jjab079, 33891004 PMC8675328

[21] Moltrasio C Romagnuolo M Tavoletti G Maronese CA Marzano AV. Pyoderma gangrenosum: pathogenetic mechanisms and their implications for treatment. Semin Immunopathol. (2025) 47:38. doi: 10.1007/s00281-025-01064-7, 41128863 PMC12549756

[22] Kotter I Zouboulis CC Vaiopoulos G . Diagnosis of Behçet’s disease: clinical characteristics, diagnostic criteria, and differential diagnoses. J Clin Med. (2021) 10:5789. doi: 10.3390/jcm10245789 33446282 PMC7809833

[23] Agarwal A Barrow W Selim MA Nicholas MW. Refractory subcutaneous Sweet syndrome treated with adalimumab. JAMA Dermatol. (2016) 152:842–4. doi: 10.1001/jamadermatol.2016.0503, 27028556

